# The rs2046210 Polymorphism Is Associated with Endometriosis Risk and Elevated Estrogen Receptor 1 Expression in the Eutopic Endometrium of Women with the Disease

**DOI:** 10.3390/biomedicines12081657

**Published:** 2024-07-25

**Authors:** Katharina Proestling, Martin Schreiber, Heidi Miedl, Quanah J. Hudson, Heinrich Husslein, Lorenz Kuessel, Manuela Gstoettner, Rene Wenzl, Iveta Yotova

**Affiliations:** Department of Obstetrics and Gynecology, Medical University of Vienna, Waehringer Guertel 18-20, 1090 Vienna, Austria; katharina.proestling@meduniwien.ac.at (K.P.); martin.schreiber@meduniwien.ac.at (M.S.); heidi.miedl@meduniwien.ac.at (H.M.); quanah.hudson@meduniwien.ac.at (Q.J.H.); heinrich.husslein@meduniwien.ac.at (H.H.); lorenz.kuessel@meduniwien.ac.at (L.K.); manuela.gstoettner@meduniwien.ac.at (M.G.); rene.wenzl@meduniwien.ac.at (R.W.)

**Keywords:** endometriosis, estrogen receptor alpha (ERα), *ESR1*, single-nucleotide variant, rs2046210

## Abstract

In this focused genetic case–control study, we analyzed two functional single-nucleotide variants (SNVs) associated with breast cancer risk (rs2046210, rs9383590) and one risk SNV for an implantation defect and infertility (rs9340799) for their association with endometriosis susceptibility, progression and *ESR1* gene regulation in endometriosis patients. The rs2046210, rs9383590 and rs9340799 SNVs were genotyped in 153 endometriosis patients and 87 control subjects with Caucasian ancestry. We analyzed the association of all SNVs with endometriosis susceptibility in all patients and in subgroups and assessed the concordance between the SNVs. Quantitative reverse transcription PCR was used to determine *ESR1* gene expression in the eutopic endometrial tissue of the controls and endometriosis patients. The heterozygous rs2046210 GA genotype was associated with significantly increased endometriosis risk, particularly in younger, leaner and infertile women and with an increased *ESR1* gene expression in the eutopic endometrium of these patients, compared to controls. The minor AA genotype of rs2046210 was identified as a potential risk factor for endometriosis progression in women with mild endometriosis. The results from this analysis indicate that rs2046210 may be a functional genetic variant associated with endometriosis development and progression.

## 1. Introduction

Endometriosis is an estrogen-driven inflammatory condition affecting about 10% of women in their reproductive age [1]. The disease is associated with chronic pelvic pain, dysmenorrhea, dyspareunia and fatigue, and often leads to infertility [2] which lowers the quality of life and mental health of affected women [2,3]. Definitive diagnosis requires surgical visualization of the lesions, which can result in a delay in diagnosis ranging from 4 to 11 years [4]. The treatment of the disease is still limited to surgical removal of the lesions and hormonal treatments with significant side effects that do not allow the women to become pregnant [5]. Endometriosis is characterized by the growth of endometrial-like tissue outside of the uterus, mainly in the peritoneal cavity. The origin of endometriosis lesions is unclear, but the most accepted hypothesis is that they arise from endometrial cells reaching the peritoneal cavity by retrograde menstruation via the fallopian tube [6,7]. In women with endometriosis, normal endometrial function is compromised due to the up-regulation of estrogen signaling mediated through elevated levels of estrogen receptor alpha (*ESR1*) expression in secretory phase endometrium, compared to controls [8]. This leads to increased estrogen-induced cell proliferation and inflammation, which, together with concurrent progesterone resistance, can promote both the development of ectopic endometriosis lesions [9,10] and reduced endometrial receptivity of women with the disease [8,11,12]. The etiology of the disease remains largely unknown, but recent research including genome-wide association studies (GWASs) has shown that the disease pathogenesis has a clear genetic component [7]. Genetic studies in twins have shown that endometriosis heritability is about 50% [13,14]. The proportion of heritability due to common genetic variation was estimated to be 26% [15], indicating that rarer variants account for the remaining heritability. Genetic variants associated with endometriosis risk have been shown to map to intergenic and intragenic non-coding regions and potentially influence the function of DNA regulatory elements and thereby the expression of a nearby gene or genes [16]. Recent large-scale GWASs of endometriosis have shown that single-nucleotide variants (SNVs) at 42 loci are associated with endometriosis risk at a genome-wide significance, with five association signals found in chromosomal region 6q25.1, which contains the *ESR1* gene [17]. Analysis of the genetic regulation and methylation at endometriosis risk loci showed that rs71575922, a high confident SNV located within 6q25.1 in an intron of the *SYNE1* gene, regulates chromatin interactions with several genes at a megabase distance [17]. Several endometriosis-risk SNVs at this locus were also identified as drivers of *ESR1* expression in endometriosis tissue [18] and blood [17].

The dysregulation of steroid hormone signaling is common in many uterine pathologies such as infertility, endometrial cancer, uterine leiomyoma and implantation failure [19]. The chromosome 6q.21 region includes several genes involved in sex-steroid hormonal signaling, such as *SYNE1*, *ESR1* and *CCDC170*, with multiple SNVs previously associated with female hormone-dependent diseases including endometrial cancer (rs79575945, rs2046210 and rs9340799) [18,20,21], breast cancer (rs2046210, rs9383590 and others) [22,23], infertility (rs9340799) [24] and recurrent implantation failure (rs9340799) [25]. Two of these SNVs (rs2046210 and rs9383590) are located in an enhancer region regulating *ESR1* gene expression and have been shown to be functional in breast cancer [22,23,26]. Despite the importance of ESR1 and estrogen signaling in endometriosis, the association of these three SNVs with endometriosis has not previously been investigated in detail. Given that endometriosis is an estrogen-dependent disease and clearly has a genetic background, we hypothesized that common SNVs functioning in *ESR1* regulation in other hormone-dependent disease conditions are shared with endometriosis. Therefore, in this study, we aimed to analyze the association of rs2046210, rs9383590 and rs9340799 with endometriosis, and their effect on the regulation of the levels of *ESR1* expression in the eutopic endometrial tissue of women with and without the disease.

## 2. Materials and Methods

### 2.1. Study Population

A total of 240 women of Caucasian ethnicity were enrolled in this study between July 2013 and July 2015. Detailed baseline characteristics of the patients are summarized in Table 1. The presence of endometriosis was determined by visual inspection during surgery and was confirmed histologically; the control group of women had no macroscopic or histological evidence of endometriosis at the time of the laparoscopy. Detailed inclusion and exclusion criteria of participating women are also described by Kuessel et al. [27]. Premenopausal women who were undergoing a laparoscopic procedure due to suspected endometriosis, chronic pelvic pain, infertility, benign adnexal masses or uterine leiomyoma were enrolled in this study. Pregnant women or women breastfeeding up to 6 months prior to the onset of the study and women who had acute inflammation, a known or suspected infectious disease, chronic autoimmune disease or malignancy were excluded from the study. Additionally, women who had received hormonal treatment within the last 3 months prior to the surgery were excluded from endometrial *ESR1* (*Estrogen Receptor alpha*) gene expression analysis. The menstrual cycle phase was determined by histopathological evaluation of the endometrial tissue. Endometriosis was classified in accordance with the revised American Fertility Society Score. In total, 68 (47.6%) endometriosis patients presented with mild (RAFS I + II of the revised American Fertility Society Score) endometriosis, and 75 (52.4%) presented with severe (RAFS III + IV) endometriosis (Table 1).

### 2.2. Sample Collection

Tissue and blood samples were collected following the protocols of the Endometriosis Marker Austria (EMMA) study, as described before [27]. Briefly, all endometrial tissue samples were collected via curettage directly before laparoscopic intervention. The tissue samples were snap-frozen directly after extraction and stored in liquid nitrogen. Blood samples were obtained directly before laparoscopic surgery using EDTA-treated tubes. All samples were collected according to the Endometriosis Phenome and Biobanking Harmonization Project guidelines [28].

### 2.3. Sample Size Calculation

The sample size for this analysis was calculated using QUANTO1_2_4-2 software. As there are no existing data for the association of the studied SNVs (single-nucleotide variants) with endometriosis and their putative function on the regulation of *ESR1* expression in the disease, we have used the additive model for determining the minimal sample size for the study to achieve a power of 0.8. In general, SNVs in complex diseases have relatively small effects on risk (typically less than 2-fold). However, as we hypothesized that rs2046210, rs9383590 and rs9340799 are functional and are involved in the regulation of *ESR1* expression, we assumed that—if disease-associated—the effect of these SNVs on disease risk will be large, i.e., equal to or greater than 2. Therefore, we used a genetic effect range of 2.0 for calculating the sample size for this study. The results of our sample size calculation showed a minimum of 109 cases and 55 controls for rs2046210, and a minimum of 110 cases and 55 controls for rs9340799. Therefore, including 153 patients and 87 controls in our study seemed to be more than sufficient. Due to the low allele frequency of rs9383590, the power for this SNV in our study is very low.

### 2.4. DNA Isolation and SNV Genotyping

For genotyping, the genomic DNA was extracted from peripheral lymphocytes using the QIAamp DNA Blood Midi kit (Qiagen, Venlo, The Netherlands) and stored at −80 °C. The SNVs were genotyped on a CFX96 real-time PCR instrument (BioRad, Vienna, Austria) as described [23]. The following assays from Applied Biosystems were used: C1203423610 for rs2046210, C3047011310 for rs9383590 and C316359110 for rs9340799 (Life Technologies, Waltham, MA, USA). A total of 20 ng of genomic DNA was used for amplification in a final PCR reaction volume of 20 µL.

### 2.5. RNA Isolation and Quantitative Reverse Transcription PCR (qRT-PCR)

Endometrial tissue samples from 37 women with and 35 without endometriosis were homogenized with a Precellys 24 homogenizer (Supplementary Table S6, PEQLAB, Erlangen, Germany). Total RNA was then isolated from eutopic endometrium samples using the Agilent Absolutely Total RNA kit (Agilent, Santa Clara, CA, USA) or the InnuPrep miRNA kit (IST Innuscreen GmbH., Berlin, Germany) including DNase I treatment. Further, RNA was reverse transcribed using the SuperScript^®^ III First-Strand Synthesis Reverse Transcriptase kit and random hexamer primers (Life Sciences Advance Technology, St. Petersburg, FL, USA). We used *GAPDH* and *ESR1* TaqMan Gene Expression Assays to assess the relative expression (Hs99999905_m1 for GAPDH and Hs00174860_m1 for *ESR1*; Applied Biosystems, Waltham, MA, USA). qRT-PCR was run in duplicates in a 7500 Fast Real-Time PCR System machine (Applied Biosystems, Waltham, MA, USA) in a final volume of 10 µL. The relative expression of *ESR1* normalized to *GAPDH* expression was calculated using the standard delta-CT method for patient data analysis [29].

### 2.6. Statistical Data Analysis

The Hardy–Weinberg equilibrium was evaluated by chi-square analysis (Had2Know, 2010–2024). Linkage disequilibrium, confidence intervals and *p*-values associated with odds ratios were evaluated using R version 3.3.2 (accessed on 1 July 2019). For *ESR1* gene expression analysis, all statistical tests were performed using GraphPad Prism software (GraphPad Prism 9.0 software, La Jolla, CA, USA). The exact statistical procedures for each analysis are described in the corresponding legends of the figures and tables.

## 3. Results

### 3.1. Baseline Characteristics of the Study Population

In this clinical case–control study, we have genotyped the rs2046210, rs9383590 and rs9340799 SNVs in a cohort of 240 women of European descent, from which 87 were women without and 153 were women with endometriosis (Table 1). The mean age and the mean body mass index (BMI) were significantly lower in the endometriosis group compared to the control group (*p* = 0.048 and *p* = 0.019, respectively).

The number of women who had been pregnant (gravidity) and the number of women who had given birth with a gestational age of 24 weeks or more (parity) was significantly lower in the endometriosis group, compared to controls (*p* = 0.0007 and *p* = 0.036, respectively; Table 1). No differences were observed in terms of overall fertility and cycle phase distribution between women with and without the disease (Table 1).

### 3.2. Distribution of rs2046210, rs9383590 and rs9340799 SNV Genotypes

**Figure 1:**
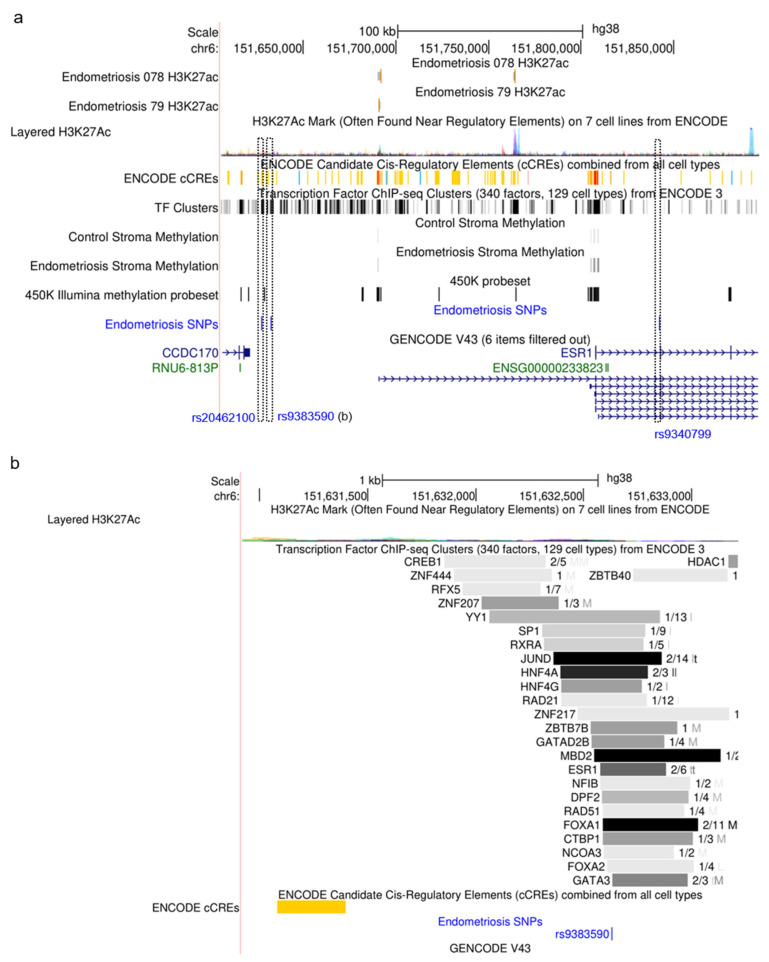
(**a**). The SNVs (single-nucleotide variants) rs2046210, rs9383590 and rs9340799 are not associated with known gene regulatory regions in endometriosis. The location of the three SNVs is plotted using USCS genomic browser tracks annotated in the hg38 build. The tracks for changes in DNA methylation in endometrial stroma cells [30] and changes in H3K27 acetylation in tissue samples of endometriosis patients and controls [31] are plotted using experimental datasets in GO and used as marks for the identification of active tissue-specific enhancers in endometriosis. The zoom into the genetic region around rs9383590 presented (**b**) is marked with a black dashed line. (**b**). Transcription factor binding sites identified by chromatin immunoprecipitation DNA-sequencing in 129 ENCODE human cell lines overlap rs9383590.

The three SNVs are located on chromosome 6 upstream and within the human *ESR1* gene. Based on the genome browser database (build 38), rs2046210 and rs9383590 are located between putative distal enhancer regions 5’ to the *ESR1* gene approximately 29 kb upstream of *ESR1* exon 1. The physical distance between rs2046210 and rs9383590 is 5399 bp, with rs9383590 being closer to the *ESR1* gene. The rs9340799 SNV is situated in *ESR1* intron 1 (Figure 1a). There was no association of any of the SNVs with changes in DNA methylation in endometriosis compared to the controls. Additionally, transcription factor (TF) chromatin immunoprecipitation-based DNA-sequencing (ChIP-seq) data from 129 different cell types showed that only rs9383590 is associated with diverse transcription factor binding, suggesting that this genomic region can act as an enhancer in different tissue types (Figure 1b). However, there is no experimental data available for the putative enhancer function of the rs9383590-associated genomic region in endometriosis.

**Figure 2:**
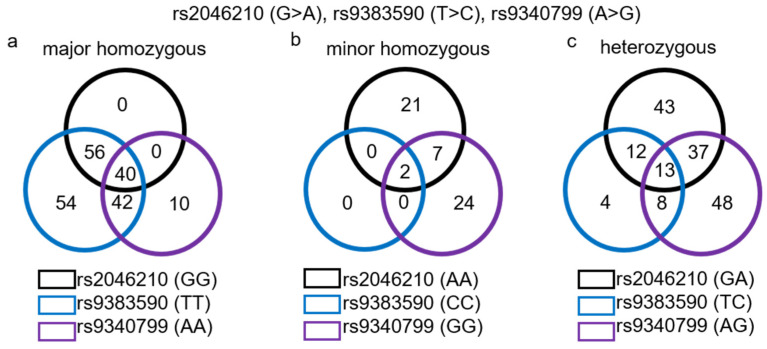
Venn diagrams showing the number of patients with co-occurrence of the three single-nucleotide variants (rs2046210, rs9383590 and rs9340799) either in their homozygous major genotype: GG for rs2046210, TT for rs9383590 and AA for rs9340799 (**a**), in their homozygous minor genotype: AA for rs2046210, CC for rs9383590 and GG for rs9340799 (**b**) or in their heterozygous genotype: GA for rs2046210, TC for rs9383590 and AG for rs9340799 (**c**).

The genotype frequencies for each SNV relative to the clinical characteristics are given in the Supplementary Tables: rs2046210 (Table S1), rs9383590 (Table S2) and rs9340799 (Table S3). In our study population, all three SNVs were in linkage disequilibrium with each other. The rs2046210 and rs9383590 SNVs were in almost complete linkage disequilibrium (D’ = 0.9988785; *p*~0), while rs2046210 and rs9340799 (D’ = 0.118977; *p* = 0.0135) and rs9383590 and rs9340799 (D’ = 0.4232486; *p* = 0.000025) also showed significant linkage disequilibrium. The frequencies of the minor allele (MAFs) for the three SNVs were similar to the global MAFs reported for the Caucasian population by the NCBI allele frequency aggregator (www.ncbi.nlm.nih.gov/snp/docs/gsr/alfa/, accessed on 2 March 2022). The MAF for the rs2046210 A allele was 0.328 for women without endometriosis and 0.3733 for women with endometriosis. The MAF for the rs9383590 C allele was 0.086 for women without and 0.088 for women with endometriosis. The MAF for the rs9340799 G allele was 0.3895 for women without and 0.3624 for women with endometriosis. The frequencies for GG, GA and AA genotypes of rs2046210 were 0.494, 0.356 and 0.149 for women without endometriosis and 0.373, 0.507 and 0.120 for women with the disease (Table S1). For rs9383590, the frequencies of the genotypes TT, TC and CC were 0.839, 0.149 and 0.01 for women without endometriosis and 0.83, 0.163 and 0.007 for women with the disease (Table S2). For rs9340799, the frequencies for genotypes AA, AG and GG were 0.360, 0.500 and 0.140 for women without and 0.423, 0.430 and 0.148 for women with endometriosis (Table S3). The populations of women without and with endometriosis were in Hardy–Weinberg equilibrium for all three SNVs. The *p*-values for the population of women without endometriosis were *p* = 0.0745 for rs204610, *p* = 0.6305 for rs9383590 and *p* = 0.6342 for rs9340799, and the *p*-values for the population of women with the disease were *p* = 0.3104 for rs204610, *p* = 0.8783 for rs9383590 and *p* = 0.389 for rs9340799. The major homozygous genotype for the three SNVs co-occurred in 40 women (Figure 2a); in addition, the major rs2046210 GG and rs9383590 TT genotypes co-occurred in 56 women, and the major rs9383590 TT and rs9340799 AA genotype co-occurred in 42 women. The major rs9340799 AA and major rs2046210 GG genotypes were not found in addition to the 40 women in a single combination in our study population (Figure 2a). The homozygous minor genotype of the three SNVs was found together only in 2 women, and in addition, the co-occurrence of the rs2046210 (AA) and rs9340799 (GG) minor genotypes was found in 7 women (Figure 2b). The heterozygous genotypes for the three SNVs co-occurred in 13 women, and in addition, the rs2046210 (GA) and rs9340799 (AG) co-occurred in 37, the rs2046210 (GA) and rs9383590 (TC) in 12 and the rs9340799 (AG) and rs9383590 (TC) in 8 women, respectively (Figure 2c).

### 3.3. Association of rs2046210, rs9383590 and rs9340799 SNVs with Endometriosis Susceptibility

To assess the association of each of the analyzed SNVs with endometriosis susceptibility, odds ratios (ORs), 95% confidence intervals (CIs) and *p*-values were first determined using recessive, dominant, log-additive and overdominant models and different allele comparisons, without adjustment for additional variables such as age, BMI or the analyzed SNVs (Table 2). This analysis showed significant association of the heterozygous rs2046210 GA genotype with endometriosis risk (OR = 1.88; CI = 1.05 to 3.36; *p* = 0.030) in a dominant model excluding the homozygous rare AA genotype. The dominant model including the homozygous rare AA genotype similarly showed a tendency for association with an increased endometriosis risk compared to the GG genotype (OR = 1.64; CI = 0.96 to 2.81; *p* = 0.069). Interestingly, in the overdominant model, the women carrying the heterozygous GA genotype show significantly increased endometriosis risk compared to women with homozygous GG or AA genotypes (OR = 1.86; CI = 1.08 to 3.19; *p* = 0.024). Indeed, the AA genotype tends to be associated with a reduced susceptibility for endometriosis compared to the GA genotype (OR = 0.56; CI = 0.25 to 1.32; *p* = 0.159). None of the remaining genotypes or alleles of rs2046210 and none of the genotypes or alleles of either rs9383590 or rs9340799 were associated with a significantly increased risk for endometriosis.

### 3.4. Effect of rs9383590, rs9340799, Woman’s Age and BMI on rs2046210-Associated Increase of Endometriosis Risk

To test whether either rs9383590 or rs9340799 could modify the association of rs2046210 with endometriosis risk, we determined the ORs, 95%CIs and the *p*-values for the rs2046210 genotypes and alleles after adjustment for either rs9383590 or rs9340799 genotypes (Table 3). The results from this analysis demonstrated that an adjustment for the rs9383590 genotype did not influence the association of rs2046210 (indicated by ORs greater than one) with endometriosis risk, showing that the increased susceptibility for endometriosis in women with rs2046210 GA vs. GG, GA + AA vs. GG and GA vs. GG + AA genotypes is independent of rs9383590. Similar results were seen after adjustment for the rs9340799 genotypes (Table 3).

Further, we analyzed the effects of women’s ages and BMIs on rs2046210-associated endometriosis susceptibility. The results from this analysis (Table 4) showed that both analyzed patient characteristics do not modify the association between rs2046210 and increased endometriosis risk. Overall, the adjustment of the rs2046210 associated endometriosis susceptibility for either rs9383590, rs9340799, age or BMI had only minor effects on the observed association of rs2046210 with endometriosis risk.

### 3.5. Stronger Association of rs2046210 GA Genotype with Endometriosis Susceptibility in Subpopulation of Younger, Leaner and Infertile Women

Subgroup analysis stratified by selected variables related to women’s age, BMI, endometriosis disease stage, fertility, gravidity and parity status showed that the association of the GA or GA + AA genotype of rs2046210 with endometriosis risk increased in women with a BMI lower than 25, an age lower than 35 years, in infertile women and in patients with mild endometriosis (RAFSI + II) compared to unselected patients (Table 2 and Table 5). The OR for women under 35 years was 3.54 (CI = 1.47 to 8.60, *p* = 0.004) for GA vs. GG, 2.63 (CI = 1.21 to 5.70, *p* = 0.015) for GA + AA vs. GG and 3.03 (CI = 1.35 to 7.01, *p* = 0.006) for GA vs. GG + AA. For women with a BMI lower than 25, the OR was 2.27 (CI = 1.12 to 4.63, *p* = 0.018) for GA vs. GG, 2.19 (CI = 1.12 to 4.29, *p* = 0.022) for GA + AA vs. GG and 2.01 (CI = 1.03 to 3.97, *p* = 0.038) for GA vs. GG + AA (Table 5). The ORs for rs2046210 were also considerably elevated in infertile women with GA vs. GG (OR = 3.30, CI= 1.24 to 8.91, *p* = 0.016), GA + AA vs. GG (OR = 2.96, CI = 1.22 to 7.22, *p* = 0.013) and in GA vs. GG + AA (OR = 2.79, CI = 1.10 to 7.24, *p* = 0.022) comparisons. In patients suffering from mild endometriosis (RAFS I + II) with the GA vs. GG genotype, the OR was increased to 2.24 (CI 1.11 to 4.51, *p*= 0.020), and with the GA vs. GG + AA genotype, it increased to 2.41 (1.24 to 4.67, *p* = 0.007) (Table 5). In women with mild endometriosis, the OR for rs2046210 GA + AA vs. GG was also elevated at borderline significance compared to unselected patients (OR = 1.78, CI = 0.92 to 3.48, *p* = 0.084, Table 5). However, using the dominant model in patient subgroups stratified by stage, BMI and age, neither rs9383590 (Table S4) nor rs9340799 (Table S5) were associated with endometriosis risk.

### 3.6. The AA Genotype of rs2046210 Might Be Associated with Increased Risk of Progressive Endometriosis

Further, we have performed analysis to evaluate the association of rs2046210 with an increased risk of progressive endometriosis, defining either GA or AA genotypes as risk-associated genetic factors. The results from this analysis show that women with mild endometriosis (RAFS I + II) carrying the AA genotype seemed to be at higher risk of developing severe endometriosis (RAFS III + IV), compared to women with the GA genotype. The OR for women with progression to severe endometriosis was 3.07 (CI = 0.91 to 11.94, *p* = 0.068) for AA vs. GA, 2.62 (CI = 0.81 to 9.91, *p* = 0.139) for AA vs. GA + GG and 0.61 (CI = 0.31 to 1.20, *p* = 0.154) for the GA vs. GG + AA genotype (Table 6).

### 3.7. The GA Genotype of rs2046210 Is Associated with the Regulation of the Levels of ESR1 Expression

**Figure 3:**
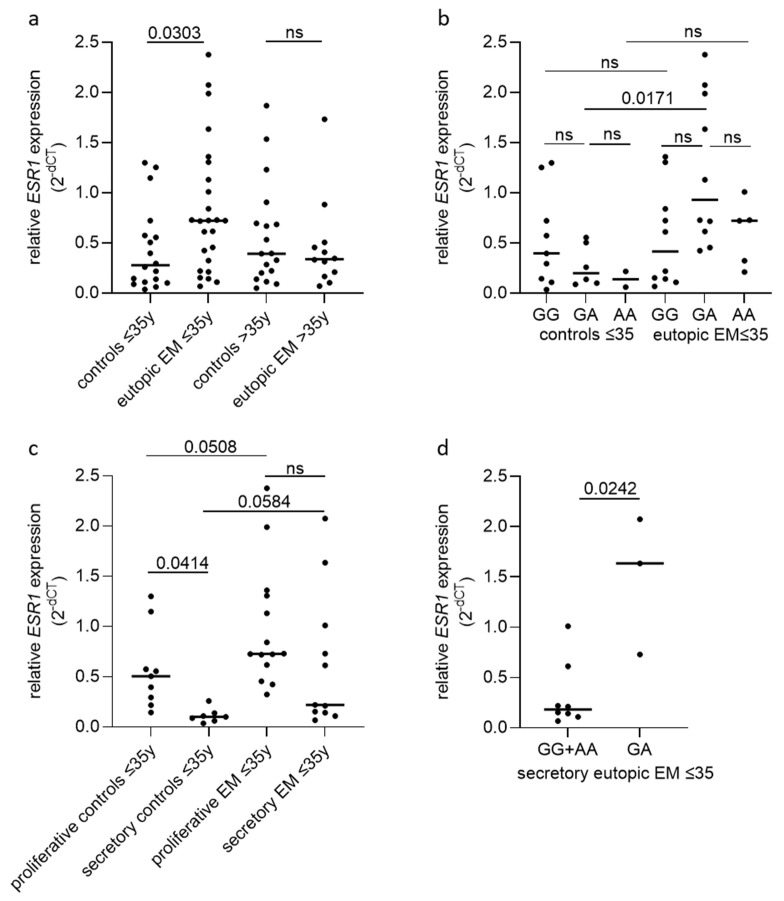
Association of rs2046210 with endometrial gene expression of *ESR1* in women with and without endometriosis. (**a**). Relative *ESR1* gene expression from tissues of women younger than 35 years with endometriosis (n = 25) is significantly increased compared to women without endometriosis (n = 18). Relative *ESR1* gene expression between women with (n = 12) and without (n = 18) endometriosis does not differ in women older than 35 years. (**b**). *ESR1* gene expression is significantly increased in women with endometriosis younger than 35 years with rs2046210 GA genotype (n = 10) compared to according controls (n = 6). No difference in relative *ESR1* gene expression between cases (n = 10 or n = 5) and controls (n = 9 or n = 2) is seen in young women with homozygous major or minor rs2046210 genotype, respectively. (**c**). In women without endometriosis younger than 35 years, endometrial *ESR1* gene expression is significantly reduced during secretory phase of menstrual cycle (n = 7) compared to proliferative phase (n = 9). In women with endometriosis younger than 35 years, *ESR1* gene expression does not significantly differ between proliferative (n = 14) and secretory (n = 11) phase. (**d**). During secretory phase, women with endometriosis who are younger than 35 years and carry GA rs2046210 genotype (n = 3) show an increase in levels of *ESR1* gene expression, compared to women with homozygous (GG + AA) rs2046210 genotypes (n = 8). *ESR1* gene expression in this figure was normalized to levels of GAPDH expression, and data are presented as scatter dot plots including median relative expression levels for each group. Data were analyzed by Kruskal–Wallis test adjusted for multiple testing using Dunn’s multiple comparisons test (**a**–**c**) or Mann–Whitney test (**d**). Adjusted *p*-values (adjp) < 0.05 were considered significant, with non-significant differences indicated by ns. Controls: endometrial tissue of women without endometriosis, eutop: endometrial tissues from women with endometriosis, >35 y: women older than 35 years, ≤35 y: women younger or equal to 35 years.

To test whether the rs2046210 GA genotype is associated with changes in the levels of *ESR1* expression, we have performed qRT-PCR to evaluate the differences in the levels of expression of the gene in eutopic tissue samples of women with and without endometriosis in a cohort of n = 72 (Supplementary Table S6). As the OR for the rs2046210 genotype GA vs. GG + AA was considerably elevated in patients aged under 35 years (Table 5), we first evaluated the differences in the levels of the ERS1 gene expression between women with and without endometriosis in this subgroup. The results from this analysis showed a 2.6-fold increase in *ESR1* mRNA levels (2.6 median increase; adjp = 0.0303) in the eutopic endometrium of women with endometriosis, compared to controls. This upregulation of *ESR1* gene expression in women with endometriosis was observed only in women aged under 35 years and not in women aged over 35 years (Figure 3a). Therefore, we have further analyzed the differences in the levels of *ESR1* expression only in young women (age ≤ 35). This analysis showed that the increased *ESR1* expression is seen in young women with endometriosis with rs2046210 GA but not with GG or AA genotypes (Figure 3b). Among the subjects with the GA genotype and an age ≤ 35 years, the levels of *ESR1* expression in endometriosis patients were 4.66-fold higher (adjp = 0.0171) than in the controls (Figure 3b).

The endometrium is a dynamic tissue whose growth is regulated by sex-steroid hormones. During a normal menstrual cycle, the endometrial levels of *ESR1* increase in the proliferative phase in response to estrogen and dramatically decrease during the window of implantation in response to progesterone in the mid-secretory phase endometrium [32]. The results shown in Figure 3c confirmed the significant downregulation of *ESR1* levels to 20.1% in the secretory phase endometrium of women without endometriosis (adjp = 0.0414). However, in the endometrium of women with endometriosis, the regulation of *ESR1* expression during the menstrual cycle seemed to be disturbed by a lack of significant downregulation of the receptor levels in the secretory phase endometrium (Figure 3c). A tendency of increased *ESR1* expression was also seen in the endometrial tissues of young women with endometriosis compared to women without endometriosis in the proliferative (1.44-fold increase; adjp = 0.0508) and secretory (2.2-fold increase; adjp = 0.0584) menstrual cycle phases (Figure 3c). Further subgroup analysis based on the rs2046210 genotype in the secretory phase endometriosis tissues of young women with the disease showed that the GA genotype is associated with a significant 8.9-fold up-regulation of *ESR1* expression (*p* = 0.0242), compared to GG + AA genotype using the overdominant model (Figure 3d).

## 4. Discussion

This genetic case–control study evaluated the association of three SNVs with endometriosis risk and with the regulation of the levels of *ESR1* expression in the eutopic endometrial tissue of women with and without endometriosis. Two of the analyzed SNVs were functional breast cancer risk SNVs located upstream of the *ESR1* gene (rs2046210, rs9383590) [22,23], and one an intronic *ESR1* SNV (rs9340799), previously associated with infertility and an implantation defect [24,25]. The results showed that the heterozygous GA genotype of rs2046210 is associated with an increased risk of endometriosis over the GG-major and AA-minor genotypes. The elevated odds ratios with respect to the endometriosis risk of the rs2046210 genotypes were not substantially changed after correction for rs9383590 and rs9340799, suggesting that the genotype of the two SNVs does not affect the risk associated with rs2046210. On the other hand, the AA genotype behaved as a somewhat “protective factor” for the disease, leading to a reduction in the odds ratios in the dominant model, compared to those for the GA genotype using the overdominant model after correction for rs9340799. In contrast to rs2046210, rs9383590 and rs9340799 were not associated with disease risk. The significant association of the GA genotype with an increased risk of endometriosis using the overdominant model remained elevated after an overall adjustment for age but not for BMI where the odds ratio tended to be slightly decreased. However, further subgroup analysis showed that rs2046210 is associated with increased endometriosis risk in younger and leaner women.

Several epidemiological and population studies on risk factors for endometriosis have shown an inverse association between BMI and endometriosis risk, and reduced BMI was suggested to mediate the genetic susceptibility to the disease [33]. The results from this study endorsed these earlier findings. Nevertheless, it is difficult to confirm that the impact of rs2046210 and BMI on endometriosis susceptibility truly represents the onset of disease in women at a younger age, as it is not possible to identify the precise time point at which endometriosis first appeared in a patient. The onset of menstruation is initiated by an increased amplitude of estrogen exposure to tissues. [34]. As we have shown that the rs2046210 heterozygous genotype is associated with increased levels of *ESR1* expression in young women, one can speculate that those women, if adolescent, will present with an increased tissue sensitivity to the hormone, and therefore, the rs2046210 can have some effect on the onset of menstruation. Menarche is a complex phenomenon that is influenced by genetic and environmental factors [34]. Genome-wide association studies have reported that the early age of menarche is associated with SNVs at several gene loci, including *ESR1* [35] and that candidate genes for the age of menarche are associated with an increased risk of developing endometriosis [36]. Compared to late menarche, early menarche was found to be associated with a 22% higher risk for endometriosis [37]. Therefore, future studies can shed light on whether the increased genetic risk of developing endometriosis for women carrying the GA genotype of rs2046210 is also due to the effect of this functional SNV on the age of menarche.

The GA genotype of rs2046210 was also strongly associated with a high risk for mild endometriosis (rAFS I and II) and in infertile women, indicating that this SNV, which is located outside of known endometriosis susceptibility signals, may act as an additional genetic factor increasing the risk of endometriosis in infertile women. The causes for endometriosis-associated infertility may range from impaired endometrial receptivity due to endocrine abnormalities to immunological disturbance and fibrosis [38]. For example, in the endometrium of women with endometriosis, the regulation of *ESR1* expression during the menstrual cycle is disturbed with higher levels of receptor expression seen in the secretory phase, compared to controls [8,38]. These higher levels of ESR1 result in enhanced estrogenic activity and proliferation, which impact the endometrial function and receptivity. Our data confirmed this finding and additionally showed that the high levels of *ESR1* expression are associated with the GA genotype of rs2046210. Although, the genetic background of endometriosis-associated infertility was extensively studied and several SNVs were identified within or close to genes involved in the regulation of estrogen signaling (*CYP19* [39], *ESR1* [24]), steroid hormone production ((*LHR*, *LH*) [40]) and inflammation ((*MUC2*) [41]), we report for the first time that rs2046210 is associated with this condition. In contrast to ER-negative breast cancer, where the GA + AA genotype of rs2046210 was shown to be associated with a downregulation of the levels of *ESR1* expression [22] in adjacent normal tissue, we found that significantly higher levels of *ESR1* expression in eutopic endometrial tissue of younger women with endometriosis are associated with the GA + AA genotype of the SNV. In addition, the increased endometrial ERS1 levels are known to play an important role in estrogen-mediated adhesion of endometriotic tissue to the peritoneum, the production of cytokines, prostaglandins and growth factors important for neoangiogenic and local inflammatory responses supporting the survival and the growth of the ectopic lesion [42]. These alterations in estrogens and estrogen receptor signaling are in accordance with our observation that the GA phenotype of rs2046210 has an effect on the susceptibility to low-grade (rAFI + II) endometriosis. These data also suggest that rs2046210 is a common genetic variant in which the minor A allele impacts the susceptibility to both pathological conditions, but on the opposite side, disease-type specifically impacts *ESR1* expression. When established, endometriosis lesions are characterized by estrogen dominance caused by the local estrogen production and altered ER signaling, presenting an overexpression of ESR2 and a downregulation of ESR1 [43,44]. In our study, the AA genotype was neither associated with an increased endometriosis risk nor with the regulation of *ESR1* expression in the endometrial tissue of young and leaner women with mild endometriosis. However, we showed that the AA genotype is a putative genetic risk factor for endometriosis progression in women (OR 3.07; CI 0.91–11.94) most probably by genetic mechanisms supporting the downregulation of *ESR1* receptor expression in women with minimal and mild endometriosis in ectopic lesions.

Although this study was performed on a well-characterized study cohort, with a clinically very well-characterized control group, the main limitation of this SNV analysis is the relatively small number of enrolled subjects. Therefore, further analysis using a large study population is needed to validate and further explore the clinical relevance of rs2046210 in endometriosis.

Overall, we have shown that rs2046210 is genetically predisposed to low-grade endometriosis, particularly in younger, leaner and infertile women, and can contribute to changes in the levels of endometrial *ESR1* expression. In addition, women with mild endometriosis carrying the minor AA genotype of rs2046210 might be at a higher risk of developing severe endometriosis. The reports for the association of other sex hormone candidate gene polymorphisms with endometriosis risk and their functionality in the pathogenesis of endometriosis [45,46,47] show that the genetic background of altered estrogen signaling and production in endometriosis development is a complex genetic trait not restricted to SNVs on the genetic region of *ESR1* on 6q.21. However, our results highlight the importance of evaluating the association of SNVs with known biological function in different pathological conditions where this functional genotype may contribute to the disease phenotype.

## Figures and Tables

**Table 1:** Clinical characteristics of the study population.

	Total (n = 240)		Controls (n = 87)		Endometriosis (n = 153)		
	Total	(%)	n	(%)	n	(%)	*p*-Value
**Age**	240		35.0 ± 7.1		33.2 ± 6.7		0.048 ^†^
Age 18–34.9	128	53.3	41	47.1	87	56.9	0.178 ^‡^
Age 35–50	112	46.7	46	52.9	66	43.1	
**BMI**	240		25.0 ± 5.9		23.4 ± 4.5		**0.019 ^†^**
BMI < 25	162	67.5	55	63.2	107	69.9	0.317 ^‡^
BMI > 25	78	32.5	32	36.8	46	30.1	
**Cycle Phase**	212		78		134		
proliferative	110	51.9	34	43.6	76	56.7	0.087 ^‡^
secretory	102	48.1	44	56.4	58	43.3	
na	28	11.7	9	10.3	19	12.4	
**RAFS**							
low (RAFS I + II)					68	47.6	
high (RAFS III + IV)					75	52.5	
na					10	6.5	
**Fertility Status**	141		57		84		
fertile	51	36.2	22	38.6	29	34.5	0.721 ^‡^
infertile	90	63.8	35	61.4	55	65.5	
na	99	41.3	30	34.5	69	45.1	
**Gravidity**	236		86		150		
0	125	53.0	33	38.4	92	61.3	**7.33 × 10^−4 ‡^**
>0	111	47.0	53	61.6	58	38.7	
na	4	1.7	1	1.2	3	2.0	
**Parity**	233		86		147		
0	166	71.2	54	62.8	112	76.2	**0.036 ^‡^**
>0	67	28.8	32	37.2	35	23.8	
na	7	2.9	1	1.2	6	3.9	

^†^ *t*-test, ^‡^ Fisher’s exact test, age and BMI (body mass index) are presented as the mean ± SD; the other variables as presented as the number (%). Na, not assessed; RAFS, revised American Fertility Society Score. Statistically significant differences are highlighted.

**Table 2:** Association of SNVs in *ESR1* with endometriosis risk.

rs2046210			rs9383590			rs9340799		
Genotypes or Alleles	OR (95% CI)	*p*- Value	Genotypes or Alleles	OR (95% CI)	*p*- Value	Genotypes or Alleles	OR (95% CI)	*p*- Value
AA vs. GG	1.06 (0.47 to 2.45)	0.919	CC vs. TT	0.60 (0.02 to 23.64)	0.764	GG vs. AA	0.90 (0.40 to 2.11)	0.753
AA vs. GA	0.56 (0.25 to 1.32)	0.159	CC vs. TC	0.54 (0.01 to 22.69)	0.764	GG vs. AG	1.23 (0.55 to 2.82)	0.618
AA vs. GG + GA (recessive model)	0.78 (0.36 to 1.71)	0.520	CC vs. TT + TC	0.59 (0.02 to 23.23)	0.765	GG vs. AA + AG	1.07 (0.50 to 2.35)	0.924
GA vs. GG (dominant model without homozygote rare)	**1.88 (1.05 to 3.36)**	**0.030**	TC vs. TT	1.10 (0.53 to 2.36)	0.783	AG vs. AA	0.73 (0.41 to 1.31)	0.275
GA + AA vs. GG (dominant model)	1.64 (0.96 to 2.81)	0.069	TC + CC vs. TT	1.07 (0.52 to 2.24)	0.929	AG + GG vs. AA	0.77 (0.44 to 1.33)	0.372
A vs. G (allele frequency model)	1.22 (0.83 to 1.82)	0.318	C vs. T	1.03 (0.53 to 2.04)	0.933	G vs. A	0.89 (0.61 to 1.31)	0.587
GA vs. GG + AA (overdominant model)	**1.86 (1.08 to 3.19)**	**0.024**	TC vs. TT + CC	0.95 (0.46 to 2.00)	0.928	AG vs. AA + GG	0.73 (0.43 to 1.24)	0.249

Analyses of cases vs. controls of the indicated genotypes or alleles. OR, odds ratio; 95% CI, 95% confidence interval. Significant associations are highlighted in bold.

**Table 3:** The association of rs2046210 with endometriosis risk after adjustment to rs9383590 or rs9340799.

rs2046210	Unadjusted		Adjusted for rs9383590		Adjusted for rs9340799	
Genotypes or Alleles	OR (95% CI)	*p*-Value	OR (95% CI)	*p*-Value	OR (95% CI)	*p*-Value
AA vs. GG + GA	0.78 (0.36 to 1.71)	0.520	0.72 (0.31 to 1.66)	0.447	0.75 (0.34 to 1.65)	0.476
GA vs. GG	**1.88 (1.05 to 3.36)**	**0.030**	1.78 (0.96 to 3.31)	0.064	**1.99 (1.11 to 3.55)**	**0.020**
GA + AA vs. GG	1.64 (0.96 to 2.81)	0.069	1.78 (0.99 to 3.2)	0.054	**1.71 (1.00–2.95)**	**0.050**
A vs. G	1.22 (0.83 to 1.82)	0.318	1.27 (0.81 to 1.97)	0.292	1.24 (0.84 to 1.86)	0.280
GA vs. GG + AA	**1.86 (1.08 to 3.19)**	**0.024**	**1.92 (1.10 to 3.35)**	**0.021**	**1.97 (1.13 to 3.41)**	**0.015**

Analyses of endometriosis cases vs. controls of the indicated rs2046210 genotypes or alleles. Analyses were conducted in all subjects unadjusted, or adjusted for the rs9383590 or the rs9340799 genotype, as indicated. OR, odds ratio; 95% CI, 95% confidence interval. Significant associations are highlighted in bold.

**Table 4:** The association of rs2046210 with endometriosis risk after adjustment to age and BMI.

rs2046210	Unadjusted		Adjusted for Age		Adjusted for BMI	
Genotypes or Alleles	OR (95% CI)	*p*-Value	OR (95% CI)	*p*-Value	OR (95% CI)	*p*-Value
AA vs. GG + GA	0.78 (0.36 to 1.71)	0.520	0.7 (0.32 to 1.53)	0.379	0.94 (0.42 to 2.1)	0.886
GA vs. GG	**1.88 (1.05 to 3.36)**	**0.030**	**1.79 (1.00 to 3.21)**	**0.049**	**1.82 (1.02 to 3.26)**	**0.042**
GA + AA vs. GG	1.64 (0.96 to 2.81)	0.069	1.54 (0.90 to 2.65)	0.117	1.66 (0.96 to 2.86)	0.068
A vs. G	1.22 (0.83 to 1.82)	0.318	1.15 (0.77 to 1.72)	0.480	1.29 (0.86 to 1.92)	0.209
GA vs. GG + AA	**1.86 (1.08 to 3.19)**	**0.024**	**1.81 (1.05 to 3.14)**	**0.031**	1.72 (0.99 to 2.99)	0.053

Analyses of endometriosis cases vs. controls of the indicated rs2046210 genotypes or alleles. Analyses were conducted in all subjects unadjusted, or adjusted for age or BMI (body mass index), as indicated. OR, odds ratio; 95% CI, 95% confidence interval. Significant associations are highlighted in bold.

**Table 5:** The association of rs2046210 with endometriosis risk in patient subpopulations.

Category	Patients		rs2046210 (GA vs. GG)		rs2046210 (GA + AA vs. GG)		rs2046210 (GA vs. GG + AA)	
	n	%	OR (95%CI)	*p*-Value	OR (95%CI)	*p*-Value	OR (95%)	*p*-Value
**Stage**								
low (RAFSI + II)	66	46.8	**2.24 (1.11 to 4.51)**	**0.020**	1.78 (0.92 to 3.48)	0.084	**2.41 (1.24 to 4.67)**	**0.007**
high (RAFS III + IV)	75	53.2	1.54 (0.78 to 3.03)	0.202	1.43 (0.73 to 2.81)	0.237	1.47 (0.78 to 2.78)	0.230
na	10	6.6						
**BMI**								
low (< 25)	161	67.9	**2.27 (1.12 to 4.63)**	**0.018**	**2.19 (1.12 to 4.29)**	**0.022**	**2.01 (1.03 to 3.97)**	**0.038**
high	76	32.1	1.21 (0.43 to 3.41)	0.702	0.93 (0.37 to 2.35)	0.909	1.32 (0.51 to 3.48)	0.556
**Age**								
18–34.9	126	53.2	**3.54 (1.47 to 8.60)**	**0.004**	**2.63 (1.21 to 5.70)**	**0.015**	**3.03 (1.35 to 7.01)**	**0.006**
35–50	111	46.8	1.05 (0.47 to 2.35)	0.920	0.98 (0.46 to 2.10)	0.924	1.11 (0.52 to 2.41)	0.774
**Fertility Status**								
fertile	43	35.5	1.67 (0.47 to 5.96)	0.447	1.11 (0.35 to 3.50)	0.889	2.14 (0.66 to 7.12)	0.203
infertile	78	64.5	**3.30 (1.24 to 8.91)**	**0.016**	**2.96 (1.22 to 7.22)**	**0.013**	**2.79 (1.10 to 7.24)**	**0.022**
na	116	49.0						
**Gravidity**								
0	104	51.5	2.41 (0.97 to 6.01)	0.057	2.14 (0.93 to 4.90)	0.072	2.13 (0.92 to 5.05)	0.083
>0	98	48.5	1.42 (0.63 to 3.19)	0.366	1.15 (0.54 to 2.45)	0.778	1.61 (0.74 to 3.49)	0.213
na	35	14.8						
**Parity**								
0	144	72.0	1.45 (0.71 to 2.95)	0.326	1.36 (0.69 to 2.66)	0.349	1.43 (0.74 to 2.78)	0.281
>0	56	28.0	2.25 (0.74 to 6.95)	0.141	1.63 (0.61 to 4.37)	0.339	2.37 (0.82 to 6.98)	0.098
na	37	15.6						

Odds ratios of the genotypes of rs2046210 in the indicated subcategories are shown. Significant associations are highlighted in bold. OR, odds ratio; 95% CI, 95% confidence interval: RAFS, revised American Fertility Society Score; BMI, bod mass index; na, not assessed.

**Table 6:** The association of rs2046210 with the potential risk of developing severe endometriosis from mild endometriosis.

	Patients		rs2046210 (AA vs. GA)		rs2046210 (AA vs. GA + GG)		rs2046210 (GA vs. GG + AA)	
	n	%	OR (95%CI)	*p*-Value	OR (95%CI)	*p*-Value	OR (95% CI)	*p*-Value
unselected patients	151	100.0	3.07 (0.91–11.94)	0.068	2.62 (0.81 to 9.91)	0.139	0.61 (0.31 to 1.20)	0.154

Odds ratios of the genotypes of rs2046210. OR, odds ratio; 95% CI, 95% confidence interval.

## Data Availability

The raw data supporting the conclusions of this article will be made available by the authors upon request.

## Supplementary Materials

The following supporting information can be downloaded at: https://www.mdpi.com/article/10.3390/biomedicines12081657/s1. Table S1: Clinical characteristics of the study population, and genotype frequencies of rs2046210. Table S2: Clinical characteristics of the study population, and genotype frequencies of rs9383590. Table S3: Clinical characteristics of the study population, and genotype frequencies of rs9340799. Table S4: Association of rs9383590 with endometriosis risk in patient subpopulations. Table S5: Association of rs9340799 with endometriosis risk in patient subpopulations. Table S6: Genotype frequencies of rs2046210 in the study population used for ESR1 gene expression. Table S7. Genotypes of rs9383590, rs9340799 and rs2046210 in women with and without endometriosis.
